# Complicated Spinal Stenosis and Spinal Deformity in Patients with Achondroplasia: Case Series and Review of the Literature

**DOI:** 10.1111/os.14246

**Published:** 2024-10-01

**Authors:** Wenyang Fu, Xianlei Gao, Xia Wang, Rongkun Xu, Shangye Li, Lianlei Wang, Xinyu Liu

**Affiliations:** ^1^ Department of Orthopedics Qilu Hospital of Shandong University Jinan China; ^2^ Qilu Hospital, Cheeloo College of Medicine Shandong University Jinan China

**Keywords:** Achondroplasia, Kyphosis, Scoliosis, Spinal Stenosis, Surgery

## Abstract

**Objective:**

Due to the low incidence of achondroplasia (Ach), there is a relative lack of research on the treatment and management of spinal complications of Ach. Characteristics and interventions for spinal complications in patients with Ach are in urgent need of investigation. This study aimed to summarize the common spinal complications in patients with Ach and the corresponding treatment strategies.

**Methods:**

This study is a retrospective case series. We retrospectively collected and analyzed Ach cases who presented to our hospital with neurological symptoms due to skeletal anomalies between February 2003 and October 2023. A total of seven patients were included, four males (57.1%) and three females (42.9%) with a mean age of 38.57 years. Patient pain/numbness visual analog scale (VAS), preoperative Oswestry disability index (ODI), development of neurological complaints, and presentation of skeletal abnormalities were collected and followed up routinely at 3, 6, 12 and 24 months postoperatively. The relevant literature was reviewed.

**Results:**

Seven patients were included in this series. The mean preoperative VAS was 4, and the mean preoperative ODI was 50.98%. All patients had concomitant spinal stenosis, four with thoracolumbar kyphosis (TLK), and one with scoliosis. Six of the seven patients underwent surgery, and one patient received conservative treatment. In the routine follow‐ups, all patients experienced satisfactory relief of symptoms. Only one of the seven patients developed a new rare lesion adjacent to the primary segments. Six months after the first surgery, a follow‐up visit revealed thoracic spinal stenosis caused by ossification of the ligamentum flavum, and his symptoms were relieved after thoracic decompression surgery.

**Conclusions:**

Ach seriously affects the skeletal development of patients and can lead to the development of spinal stenosis, spinal deformities, and other complications of the locomotor system. Surgery remains the primary treatment for complications of the musculoskeletal system. Specific surgical approaches and comprehensive, long‐term management are critical to the treatment of patients with spinal complications.

## Graphical abstract

Achondroplasia has a major impact on a patient's skeletal growth and can cause spinal stenosis, spinal abnormalities, and other locomotor system issues. Surgery is still the primary therapy for musculoskeletal system complications.

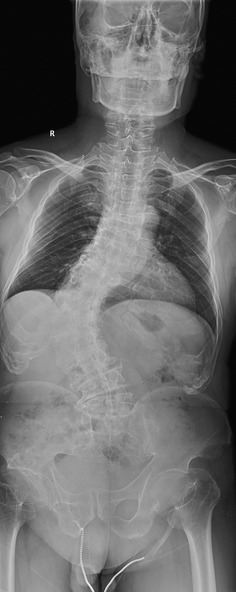

## Introduction

Achondroplasia (Ach) is an inherited disorder of skeletal development and the most common form of dwarfism.^1^, ^2^, ^3^, ^4^ The prevalence of Ach varies from 1 in 10,000 to 1 in 35,000 in different studies.^1^, ^3^, ^5^, ^6^ Clinically, it frequently manifests as a short stature malformation with a recognizable, huge head and short limbs.^6^, ^7^, ^8^ Due to impaired proliferation and differentiation of chondrocytes, patients with Ach are often present with congenital spinal stenosis and spinal deformities. Stenotic spinal canal can lead to compression of the spinal cord in the corresponding segment, resulting in neurological symptoms such as pain, numbness, sensory abnormalities, and motor deficits.^2^, ^9^, ^10^ Degenerative changes such as bulging discs, hyperplasia of bony structures, and ossification of the ligamentum flavum (OLF), a rare lesion, can exacerbate compression of the dural sac. Skeletal developmental disorders lead to complications in the spine of Ach patients along with abnormalities in the spine itself. And, patients can have multiple spinal complications at the same time or develop new complications as the disease progresses. However, due to the low prevalence of Ach, most of the current research has been limited to case reports or epidemiological studies, not involving long‐term, comprehensive, and individualized interventions for patients.

This study retrospectively collected and analyzed the clinical manifestations, imaging data, and treatment process of spinal complications in seven Ach patients with the objective to (i) summarize and analyze the occurrence and development of common spinal complications in Ach patients in conjunction with previous literature and (ii) assist in the treatment and management of spinal complications in patients with Ach. In addition, to the best of our knowledge, we report the first case of a patient with simultaneous thoracic spinal stenosis, lumbar spinal stenosis, and thoracolumbar kyphosis with postoperative ossification of the thoracic ligamentum flavum leading to symptomatic thoracic spinal stenosis.

## Methods

### Baseline Information

The study was designed as a retrospective study and was approved by the Institutional Review Board (KYll‐2021(ZM)‐058). All Ach cases presented to our institution between February 2003 and October 2023 were retrospectively reviewed. Inclusion criteria were (i) Ach patients with typical clinical features. (ii) Clinical records and radiological data were available. Exclusion criteria were (i) Ach patients without spinal complications and (ii) dwarfism due to endocrine or nutritional factors. In total 111,186 patients with spine lesions, 10 patients were diagnosed with Ach. Of the 10 patients, seven had neurological symptoms due to skeletal abnormalities and received interventions at our institution. The remaining three patients received obstetric interventions due to labor difficulties.

Of the seven ACH patients who presented with neurologic symptoms, six underwent surgical intervention. This series included four males (57.1%) and three females (42.9%).The mean age at the time of the intervention was 38.57 years old. These seven patients were diagnosed with ACH based on clinical presentation, and three of them underwent genetic testing as an aid to diagnosis.

### Patient Assessment

Preoperative visual analogue scale for pain/numbness (VAS), preoperative oswestry disability index (ODI), development of neurologic complaints, and manifestation of skeletal abnormalities were all collected from the patients. Following admission, routinely conducted X‐rays, computed tomography (CT), and magnetic resonance imaging (MRI) were performed to ascertain the etiology and appropriate therapy options. One to 7 days following surgery, patients who underwent surgery got postoperative CT scans and X‐rays. Routine follow‐up was carried out at 3, 6, 12, and 24 months after surgery.

## Results

The mean preoperative VAS was 4, and the mean preoperative ODI was 50.98%. Patients' symptoms and musculoskeletal complications were listed in Table 1, respectively. Six of the seven patients underwent surgery, and one patient's symptoms were adequately relieved with conservative treatment. In the routine follow‐ups, all patients experienced satisfactory relief of symptoms. Only one of the seven patients developed a new rare lesion adjacent to the primary segments.

**TABLE 1:** Symptoms and complications in patients with Ach.

Patient	Sex/age (years)	Symptom	Complication	Intervention	Improvement
Patient 1	M/26	Low back pain and pain and numbness in bilateral lower extremities	SS, TLK, OLF	Decompressive and corrective surgery	Yes
Patient 2	M/44	Numbness in both lower extremities with difficulty in urination and defecation	SS, scoliosis	Decompressive surgery	Yes
Patient 3	M/40	Pain in the buttocks and both lower extremities	SS	Decompressive surgery	Yes
Patient 4	F/62	Lower back pain with weakness of both lower limbs	SS	Conservative treatment	Yes
Patient 5	M/52	Low back pain and pain and numbness in bilateral lower extremities	SS, TLK	Decompressive and corrective surgery	Yes
Patient 6	F/15	Weakness of both lower limbs	SS, TLK	Decompressive and corrective surgery	Yes
Patient 7	F/32	Numbness and weakness of both lower limbs	SS, TLK	Decompressive and corrective surgery	Yes

Abbreviations: OLF, ossification of the ligamentum flavum; SS, spinal stenosis; TLK, thoracolumbar kyphosis.

### Classical Case Report

#### Case 1

A 26‐year‐old man came to our clinic with pain and discomfort in the lower back and legs for more than 1 month. The patient developed post‐exertional lumbago with pain and numbness in both lower extremities 1 month ago, with the left lower extremity being the heaviest. General physical examination revealed a short stature and stunted growth with rhizomelic limb shortening. Pressure pain on the left side of the L5, S1 spinous process, and hyperalgesia of the left anterior tibial skin were found. The muscle strength test revealed grade IV bilateral knee extension and hip extension muscles and grade IV left toe dorsiflexion muscles. The bilateral knee reflex and Achilles tendon reflex were both weaker, and the bilateral Ely test was positive. CT examination showed shortened pedicles, reduced interpedicular distance, L3/4 and L4/5 disc bulge with corresponding segmental stenosis, and L5/S1 disc herniation and thoracolumbar kyphosis. Shortened pedicles and reduced interpedicular distance demonstrate typical spinal stenosis in patients with Ach. The patient was diagnosed with lumbar disc herniation, thoracolumbar spinal stenosis, thoracolumbar kyphosis, and Ach.

Intraoperative resection of the T10‐L5 spinous process and lamina revealed hypertrophy of the ligamentum flavum, coalescence of the articular processes, and stenosis of the spinal canal. The spinal canal was subliminally enlarged, the ligamentum flavum was removed, and the canal was completely decompressed. The superior articular eminence, pedicle, and upper half of the vertebral body of L2 were removed to ensure adequate decompression. Appropriate compression was applied to correct the kyphosis deformity to reduce pressure on the patient's spinal canal contents and restore the patient's sagittal alignment. The incision healed well after the operation, and the patient's painful numbness and weakness of the lower limbs improved.

**FIGURE 1:**
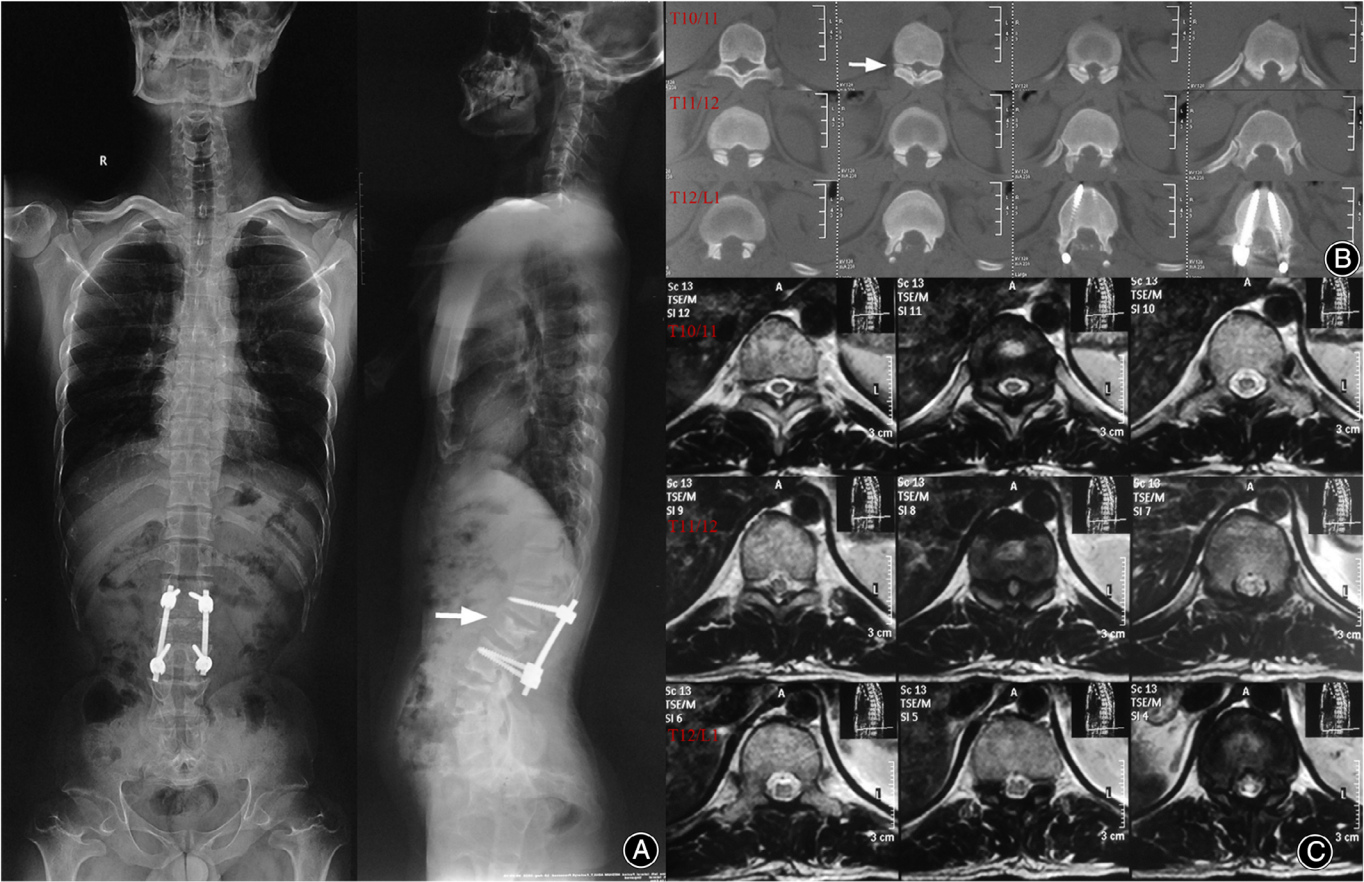
Radiographic data prior to reoperation in case 1. Lateral *x*‐ray image shows the wedge‐shaped changed L2 vertebrae (arrow in A). Axial CT shows ossification of the ligamentum flavum at the level of T10–11 (arrow in B).

The patient received regular, comprehensive follow‐ups and outcome assessments. At the 3‐month postoperative follow‐up, the patient had developed lower extremity weakness about 1 month earlier, and an X‐ray examination suggested wedge‐shaped changes in the L2 vertebral body (Figure 1A). Six months after the surgery, the patient presented to the clinic with weakness in the lower extremities. The patient developed weakness in the lower extremities 4 months ago, mainly in the left lower extremity, accompanied by numbness in the left lower extremity and a feeling of stepping on cotton in both feet. Physical examination revealed that the patient had pressure pain in the L3 spinous process and hyperalgesia in the skin of the left medial calf. Muscle strength test results indicated left hip flexor strength grade III, bilateral knee flexor strength grade 0, left foot dorsiflexor strength grade II, and right foot dorsiflexor strength grade IV. The knee reflex and Achilles tendon reflex were weak bilaterally. CT examination showed OLF at the level of T10‐11 (Figure 1B) and wedge‐shaped changes in the L2 vertebral body. MRI suggests T10/11 and T11/12 disc herniation with spinal stenosis, spinal cord degeneration, and L2 vertebral wedge deformation (Figure 1C). The patient was diagnosed with thoracic spinal stenosis.

The patient underwent posterior thoracic decompression under general anesthesia. Intraoperatively, a large amount of scarring was seen posterior to T11. The superficial scar was excised, and the T10 plate was abraded, and stenotic canal with the OLF at the level of T10/11 was seen. Considering the effects of the patient's scar from the previous surgery and OLF, combined with the abnormal development of the spinal canal in patients with Ach, the ligamentum flavum was removed, and the proximal scar was loosened and partially excised to adequately decompress the spinal canal. Postoperatively, the patient's lower extremity symptoms were relieved. Routine periodic follow‐up was performed after the second surgery. The patient experienced sustained and adequate relief of symptoms.

#### Case 2

**FIGURE 2:**
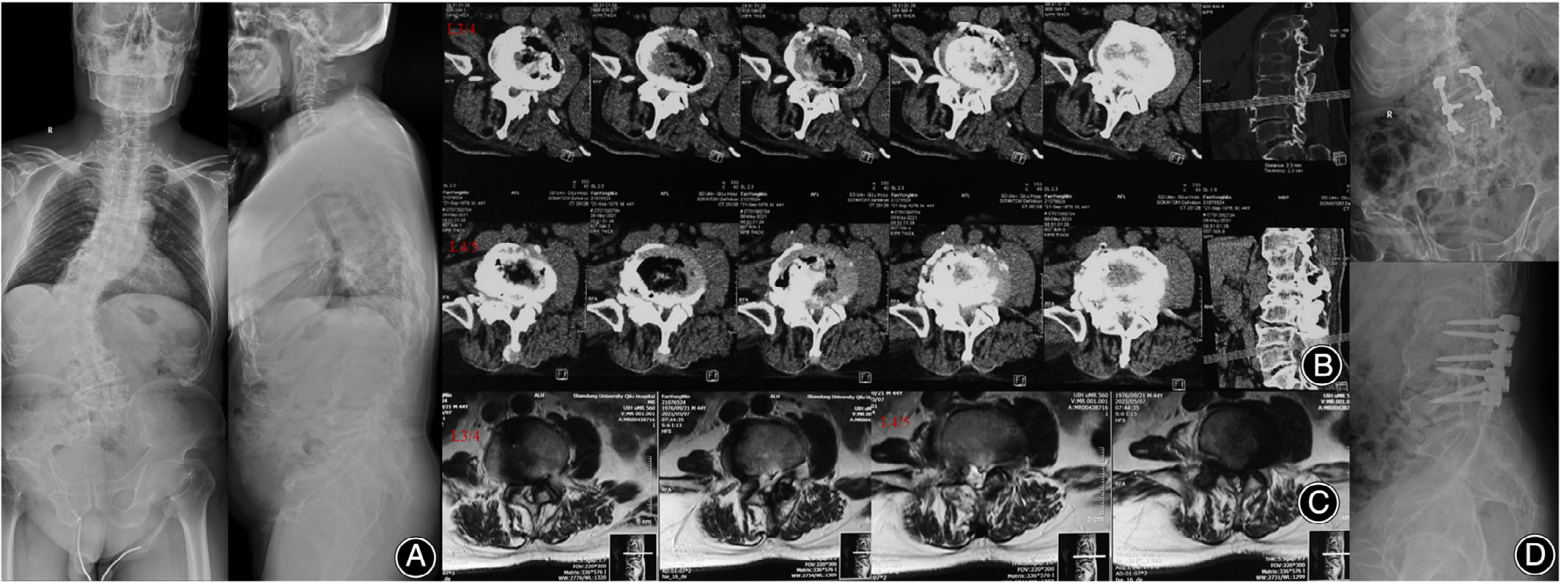
Radiographic data prior to operation in case 2. PA X‐ray image shows thoracolumbar scoliosis with a cobb angle of 65° (A). Axial CT and MR images show L4/L5 disc herniation with L3/L4 and L4/L5 segmental spinal stenosis (B, C). (D) presents the postoperative lumbar spine X‐ray image.

A 44‐year‐old male dwarf presented with chief complaints of numbness in both lower extremities for more than 1 year. The patient developed numbness in both lower extremities with no obvious cause 1 year ago, and the painful numbness was limited, mainly in the calves and soles of the feet bilaterally. The pain worsened after exertional activity and could be mildly relieved after rest. The patient also developed urinary and fecal disorders. Now the patient's numbness in both lower limbs is progressively worsening. The patient had a history of cerebral ischemia for more than 1 month, and was treated with oral aspirin, and prepared Chinese medicine with poor symptom control. The patient had a history of cervical discomfort for more than 1 month without treatment. Physical examination revealed a short physique, scoliosis deformity, and a mild claudication gait. The right toe dorsal extension muscle strength is grade IV, the remaining lower limb muscle strength did not show significant changes. The left ankle reflex was normal, whereas the right ankle reflex was slightly hyperactive. Pathological signs were not evoked bilaterally. VAS score for lower extremity numbness was 7. MRI at the local hospital suggested multiple ischemic brain foci; C3/4, C4/5, and C5/6 disc herniation; C3/4 and C4/5 spinal stenosis; L4/5 and L5/S1 disc herniation with corresponding segmental stenosis; and L3/L4 spinal stenosis, scoliosis, and lumbar dorsal myofasciitis (Figure 2C). The patient was diagnosed with lumbar disc herniation, lumbar spinal stenosis, scoliosis, cervical disc herniation, cervical spinal stenosis, cauda equina syndrome, cerebral ischemia, and Ach.

The patient had multisegmental stenosis with a history of cerebral ischemia; thus, the responsible lesion for symptoms needed to be identified for a minimally invasive and individualized surgical plan. Posterior lumbar decompression and graft fusion were carried out after surgical contraindications had been ruled out. The L3–L5 spinous process, the vertebral lamina, and the articular eminence joint were revealed. The lower edge of the L2 lamina, the L3 and L4 spinous process laminae, and the hypertrophic ligamentum flavum were removed. Postoperatively, the patient's symptoms improved, and the incision healed well.

## Discussion

Seven patients experienced adequate relief of symptoms after surgery or conservative treatment. Routine follow‐up suggested satisfactory relief of symptoms in all patients. Postoperative follow‐up suggested that one patient developed a new rare lesion in the adjacent segment to the primary segment, which has not been previously reported.

### Spinal Complications in ach Patients

Ach is an autosomal dominant disorder caused by a mutation in the short arm of chromosome 4 that affects the fibroblast growth factor receptor 3 (FGFR3) gene.^1^, ^5^ Different mutations of *FGFR3* can cause hypochondroplasia, Ach, severe Ach with developmental delay and acanthosis nigricans, thanatophoric dysplasia, and many other diseases that manifest as short stature.^3^ One of the mutations causing Ach is a glycine to arginine substitution at residue 380, leading to overexpression of FGFR3 in chondrocytes, which inhibits chondrocyte proliferation and differentiation after fetal birth through downstream signaling pathways.^1^, ^3^, ^5^ Most of the mutations causing Ach are *de novo* mutations, and almost all of them are derived from the parental chromosome.^1^, ^3^, ^4^, ^5^, ^6^, ^10^ Most studies suggest that older paternal age, especially >35 years, may be a risk factor for Ach, which may be related to the selective advantage of sperm carrying mutant *FGFR3*.^3^, ^6^, ^10^

The clinical features of Ach are shortening of the long bones, disproportionate shortening of the proximal segments, joint hyperextension, limited elbow extension and rotation, macrocephaly, midface hypoplasia, and frontal bossing.^1^, ^3^, ^4^, ^6^, ^7^, ^8^, ^9^, ^10^, ^11^, ^12^, ^13^, ^14^, ^15^ The patients reported herein largely presented with typical manifestations of Ach. The diagnosis of Ach frequently relies more on clinical signs, despite the fact that genetic diagnosis is neither expensive nor labor‐intensive.^3^, ^6^ In this report, three patients underwent DNA testing. Due to severe disorders of skeletal development, patients with Ach often exhibit multisystem or multisite complications. Hunter *et al*. summarized the probability of various common complications of Ach and the age of predisposition in a multicenter study.^2^ The spine of Ach patients often exhibits anomalies such as spinal stenosis, thoracolumbar kyphosis, and thoracolumbar scoliosis, which is attributed to the inhibition of chondrocyte proliferation and development.^16^ Spinal stenosis and skeletal abnormalities are well‐documented consequences that have a major influence on patients' quality of life. Studies by Hoover‐Fong *et al*. and Ireland *et al*. show the frequency of these issues and their clinical significance.^17^, ^18^ In this paper, seven Ach patients with corresponding symptoms and spinal complications are described. Four of these individuals showed thoracolumbar kyphosis, one had scoliosis, and all of these cases had spinal stenosis. The typical presentations in these cases align with these findings, exemplifying the clinical spectrum of Ach‐associated spinal pathologies and validating the representativeness of this series. To our knowledge, only seven articles in English have reported symptomatic spinal stenosis in patients with Ach due to OLF (Table 2). In this article, we report a rare case of Ach presenting with thoracolumbar spinal stenosis, thoracolumbar kyphosis, and symptoms of thoracic spinal stenosis caused by OLF after the first surgery.

**TABLE 2:** Cases reported involving ligamentum flavum ossification in patients with achondroplasia.

Study	Sex/age (years)	Ossification levels	Neurological status	Improvement
Suzuki *et al*. (2008)^7^	M/53	T9‐12	Intermittent claudication	Yes
Saito *et al*. (2014)^8^	M/75	L1‐4	Intermittent claudication	Yes
Cabrera‐Aldana *et al*. (2016)^32^	F/45	T3‐10	Myelopathy	Unknown
Chakraborty *et al*. (2017)^33^	F/30	T10‐12	Myelopathy below T10	Yes
Kachonkittisak *et al*. (2019)^22^	M/52	T10‐L5	Intermittent claudication + myelopathy below T10	Yes
Gokcen *et al*. (2019)^34^	M/24	T10‐L4	Intermittent claudication + myelopathy	Yes
Nanda *et al*. (2021)^35^	F/34	T9‐L5	Intermittent claudication + myelopathy	Yes

The chondrification centers of the vertebral bodies and those of the arches often fuse prematurely during the growth of the patient's spine as a result of defects of chondrocyte proliferation and maturation, leading to a relatively narrowing of the spinal canal.^12^ Abnormal cartilage and bone development often characterize the spine of patients with Ach: normal vertebral body height and width, but significant shortening of the pedicles, reduced interpedicular distance, and deeper lateral fossae.^10^, ^11^, ^12^, ^14^, ^19^ Patients may also present with a decrease in vertebral height and an increase in vertebral width at the endplate.^20^, ^21^ The average reduction in spinal canal area of 1/3–1/2, combined with degenerative changes such as disc herniation, facet joint hyperplasia, thickening of the vertebral lamina, and hypertrophy of the ligamentum flavum, result in symptomatic spinal stenosis in most patients, typically after the age of 30, considerably earlier than normal and with rapid progression.^2^, ^6^, ^9^ Owing to disorders of cranial development, patients with Ach often have an occipital foramen that is much smaller than that of a normal adult and is often positioned anteriorly, resulting in hyperextension and congenital compression of the cervical medulla.^10^ Patients may experience apnea and symptoms of nerve injury before the age of 20 if they have a foramen magnum deformity and cervical spinal stenosis. Additionally, thoracolumbar kyphosis, a common spinal complication in Ach patients, can exacerbate or induce the symptoms of thoracic spinal stenosis and lumbar spinal stenosis caused by congenital spinal developmental disorders and age‐related degenerative changes, which include walking difficulty, lower extremity pain, lower extremity sensory abnormalities, lumbar pain, and cauda equina disorder.^14^, ^15^, ^22^

Thoracolumbar kyphosis (TLK) is another common spinal complication in patients with Ach, with an incidence of up to 79%.^6^, ^9^, ^15^, ^19^ Unlike spinal stenosis, which often occurs in adult patients, thoracolumbar kyphosis can present at birth and keep developing until 4 months of age.^2^, ^6^, ^9^ In contradiction to the continued progression of spinal stenosis, the kyphosis may resolve spontaneously. Although a conclusive investigation on the incidence and progression mechanisms of TLK is lacking, Kopits *et al*. observed that older Ach patients had a much lower incidence of thoracolumbar kyphosis.^23^ As a frequent spinal complication of Ach, TLK can also induce a range of clinical symptoms. The kyphosis in infancy may result in hypotonia of the trunk, which resolves at 12–18 months of age. As mentioned above, the kyphotic deformity can also act as a triggering factor to induce patients to develop symptoms of spinal stenosis in young adulthood. Furthermore, it has been asserted that the emergence of neurological symptoms is directly related to kyphotic angles greater than 20 degrees; however, subsequent research has not confirmed this claim.^14^, ^22^ In the cases described here, four patients presented with thoracolumbar kyphosis, and their kyphosis deformities were corrected to achieve adequate decompression of the dural sac.

Another prevalent spinal deformity issue that affects Ach patients is scoliosis.^3^, ^9^, ^15^ In research with a small sample size, Kahanovitz *et al*. reported that Ach complicated scoliosis in about one‐third of patients, with Cobb angles ranging from 8° to 30°.^22^ Khan *et al*., on the contrary, found in a multicenter study that up to 60% of patients with Ach had concurrent scoliosis and 6.1% had a scoliosis angle >25°.^15^ Scoliosis is mainly distributed in the thoracolumbar segment, followed by the thoracic segment. Scoliosis generally has little association with patient‐related presentations since the majority of patients have a small Cobb angle. However, for severe scoliosis, close follow‐up observation is indispensable. The scoliosis in one of the patients in this paper occurred in the thoracolumbar segment with a Cobb angle of approximately 65° (Figure 2A), and the large scoliosis angle may have exacerbated the compression of the dural sac by the stenotic canal.

### Treatment and Management of Spinal Complications

Ach is a congenital, systemic disorder. Treatment and care for its primary manifestations and complications often persist throughout life.^6^ Traditional treatment strategies include surgery and recombinant human growth hormone (r‐hGH).The operation is known as “limb lengthening surgery” and is intended to increase the patient's height and improve the patient's quality of life. Nevertheless, the procedure is painful and can be associated with unpleasant side effects such as infection, muscle contracture, and an increased risk of fracture. Short‐term application of r‐hGH increased the height of patients, but the long‐term efficacy was not determined and the improvement of abnormal proportions of patients' body posture was not sufficient. Benefiting from the research on its pathological mechanisms, different therapeutic regimens targeting FGFR3 activation and other molecular mechanisms are being tested.^3^

Spinal complications can have a significant impact on the quality of life of Ach patients, creating significant physical and psychological challenges. Low back and leg pain in patients can be debilitating, emphasizing the need for personalized treatment to mitigate these multifaceted effects and improve quality of life.^6^, ^24^ Despite the advances in research on the etiology and treatment of Ach, surgery is still the main treatment for skeletal dysplasia, an important complication that affects individuals' physical and mental health as well as their quality of life.^3^, ^4^ Spinal stenosis being a common complication of Ach, patients often come to the clinic with neurological symptoms, and surgery is probably the only option to relieve the patient's symptoms and avoid further impairment of the spinal cord. Laminectomy is planned and carried out by a spine surgeon familiar with Ach soon after the patient presents with an indication to achieve decompression of the stenotic segment and prevent further spinal cord injury.^6^ Compared to other patients with lumbar spinal stenosis, Thommer and Van Dijk *et al*. found that the lumbar spinal canal in patients with Ach did not exhibit stenosis at the level of the lamina due to the compensation of the pseudoscalloping, and they adopted interapophyseolaminar decompression instead of routine laminectomy for the treatment of lumbar spinal stenosis complicating patients with Ach.^12^ Similar to lumbar spinal stenosis, the symptoms of thoracic spinal stenosis presenting in Ach patients can be treated with a similar surgical procedure, and satisfactory surgical outcomes have been attained in both.^12^, ^14^ The patients reported in this paper were treated with a laminectomy to ensure adequate and effective decompression.

Regardless of the fact that surgery guarantees sufficient decompression, approximately 70% of patients still report the persistence of associated symptoms at postoperative follow‐up.^4^ The spinal stenosis and deformities in patients with Ach present unique therapeutic challenges when compared to degenerative spinal disorders. Patients with Ach tend to have a younger age of onset of spinal complications due to congenital spinal stenosis, which contrasts with the gradual progression seen in patients with degenerative pathologies. The spinal stenosis pattern in Ach disease is also unique, often involving multiple levels with associated skeletal deformities, such as thoracolumbar lordosis and scoliosis, which complicate the clinical picture.^27^ These congenital deformities require early intervention to prevent rapid progression and severe dysfunction, and thus require management strategies specific to Ach patients.^17^ All patients reported in this paper who received decompression surgery had relief of their symptoms.

Ach has unique anatomical challenges, such as reduced interpedicular distance, multisegmental stenosis, and kyphotic deformities, which require specialized surgical planning and techniques. Since patients with Ach have a weak spinal dura, reduced epidural fat, which provides weaker protection of the neural component, laminectomy can often be accompanied by dural tears, nerve root injury, and other complications, all of which affect the long‐term prognosis of the patient.^11^, ^13^, ^16^ The most frequent side effects of laminectomy in Ach patients are dural rips, followed by neurological issues and surgical incision infections.^11^, ^16^ Surgeons often need to adapt standard approaches including minimally invasive surgery and customized spinal stabilization methods and may require specialized tools to accommodate the unusual spinal anatomy, ensure safe navigation, and minimize risks such as dural tears.^11^, ^13^, ^16^ Intraoperatively, precise navigation is essential to avoid complications, especially when dealing with a narrow spinal canal.^25^ Fortunately, with the aid of navigation, no surgical complications were found in these six patients at postoperative follow‐up, and all patients healed and recovered satisfactorily after the operation.

While the mechanism is unclear, the disturbed chondrogenic differentiation and proliferation in Ach patients often contribute to the presence of spinal deformities. In a cross‐sectional study, Mahomed *et al*. found a 30% prevalence of spinal deformities in patients with Ach, yet the operative rate for performing corrective surgery was only 7%.^4^ This contrast may be associated with the slight degree of deformity in most patients, the corresponding mild clinical symptoms, and the inadequate level of local medical care. Thoracolumbar kyphosis, a prevalent spinal deformity in patients with Ach, can occur in up to 90% of cases.^26^ Currently, however, there is relatively limited research available on its clinical presentation, progression, and treatment.^19^, ^28^ This may be explained by the fact that the kyphosis and associated symptoms may heal spontaneously or resolve with age, and the ambiguous definition may be another reason restricting its study.^19^, ^21^, ^23^, ^26^ The kyphosis in four of the cases reported here was apparent, and the combination of the patients' pre‐existing spinal stenosis may have aggravated the compression of the patient's spinal canal contents. In anticipation of adequate decompression, we performed correction alongside removal of the lamina, and the patients' symptoms improved substantially after surgery. Probably as a consequence of the low prevalence and small angle, scoliosis complicated by Ach patients is seldom studied as a stand‐alone problem in terms of symptoms and surgical treatment, in spite of its frequent statistical study as one of the complications of Ach.^9^, ^15^, ^16^ One of the patients reported here presented with a fairly severe thoracolumbar scoliosis deformity. But given his clinical condition and the patient's personal preferences, we did not perform corrective surgery. After decompression surgery, the patient's numbness and pain were sufficiently alleviated.

The skeletal developmental disorders of Ach are systemic, often involving the development and function of other systems as well, and require a multidisciplinary surgical solution.^2^, ^6^, ^9^ Only 23% of patients received lumbar spinal decompression in the study by Mahomed *et al*., although almost two‐thirds of patients required at least one surgery.^4^ Six of the seven patients reported in this article underwent lumbar decompression, which was closely related to the symptoms of the presenting population. Three of the seven patients underwent at least two procedures. One of the patients developed neural symptoms because of the stenosis of different segments of the spinal canal. The index segments of the third surgery were adjacent to the second surgery region, and it is possible that the second surgery promoted degenerative changes in the adjacent segment, leading to the onset of symptomatic stenosis. In the other case, postoperative OLF in the adjacent segment led to the corresponding symptoms. The initial preoperative examination did not suggest OLF; however, the postoperative examination at 6 months revealed OLF, possibly resulting from the effect of surgery on the load applied to the spine and its supporting structures, which facilitated the process of OLF. To the best of our knowledge, there are few studies investigating multiple surgeries and postoperative changes in adjacent segments in Ach patients. Karim Shafi *et al*. reported that eight of 19 Ach patients who underwent spinal surgery underwent a repeat spinal surgery during follow‐up, and four of these patients underwent a third spinal surgery; however, the report concentrated mostly on revision surgery and reoperation of non‐adjacent segments.^16^ In the other cases, the presence of thoracolumbar kyphosis after decompression has only been found in adolescent patients.^29^, ^30^ The lack of research may be attributed to the challenge of maintaining contact between different surgical units and the low incidence of the disease itself.

Due to the systemic nature of Ach and the need for multiple surgeries, prolonged, regular postoperative follow‐up and multidisciplinary care are particularly important. Postoperative care is tailored to the specific needs of Ach patients, with a focus on pain management, specialized rehabilitation programs, and vigilant monitoring of potential complications to optimize recovery and patient outcomes. A comprehensive outcome assessment through long‐term follow‐up reveals the durability of these interventions and their impact on patients' quality of life. Multidisciplinary care integrates the expertise of orthopedics, neurology, and rehabilitation to ensure a holistic approach to patient management.^18^ Emphasis is placed on educating patients and families about the surgical procedure and postoperative care, as well as developing individualized rehabilitation programs that lead to improved outcomes and functional prognosis.^17^ Innovations in remote monitoring and the use of patient‐reported outcome measures provide a basis for understanding patient experience and satisfaction, thereby guiding continuous improvement in care.^31^ These novel strategies emphasize the importance of a patient‐centered approach to managing Ach, which ultimately contributes to improved surgical outcomes and quality of life for this patient population.

### Limitations and Strengths

Our case series contributes to our understanding of spinal problems in Ach by emphasizing the need for customized surgical methods and the value of diligent postoperative monitoring. For example, one patient's treatment of thoracolumbar kyphosis with lumbar disc herniation required a nuanced approach to decompression and correction, demonstrating the vital necessity for individualized surgical planning in Ach owing to distinct anatomical characteristics. Another instance with a patient with many spinal abnormalities, including scoliosis and spinal stenosis, illustrated the complexities of establishing optimal spine alignment and function, which necessitated a comprehensive surgical and postoperative approach. Notably, the appearance of a new lesion post‐surgery in one patient, resulting in thoracic spinal stenosis, highlights the possibility of adjacent segment illness and emphasizes the importance of continuous surveillance. These cases demonstrate the multifaceted nature of spinal disease in Ach patients, the difficulty of surgical therapy, and the critical need of continuing follow‐up, providing unique insights into the specific care required for this patient population.

This study also has some shortcomings. First, this study was a single‐center, small‐sample study that included a concentrated sample of a single race and region. Second, this study evaluated the clinical scores and imaging characteristics of patients with Ach, but was unable to clarify the postoperative changes in the spine and their mechanisms in patients with Ach due to the limitation of the number of cases. Future studies should include large multicenter samples and focus on the postoperative changes in spinal alignment and the occurrence of other lesions in adjacent segments.

## Conclusion

Ach causes significant disruptions to the skeletal development of patients and can result in the development of spinal abnormalities, spinal stenosis, and other locomotor system issues. In this paper, we also report, to the best of our knowledge, the first patient with Ach presenting with symptoms of thoracic spinal stenosis secondary to OLF in the adjacent segment after the primary surgery. Individualized surgical procedures and long‐term postoperative monitoring are critical to deal with the unique spinal structures and diverse spinal complications of Ach patients.

## Funding Information

This work was supported in part by the National Natural Science Foundation of China (81874022, 82172483, and 82102522), National Key R&D Program of China (2023YFC2509700), Taishan Scholar Project of Shandong Province (tsqn202211317 and tstp20231247), Key Technology Research and Development Program of Shandong Province (2022CXGC010503 and 2022ZLGX03), Shandong Natural Science Foundation (ZR202102210113), and National High Level Hospital Clinical Research Funding (2022‐PUMCH‐D‐004).

## Conflict of Interest

The authors declare no conflicts of interest.

## Author Contributions

Wenyang Fu: methodology, investigation, formal analysis, writing—original draft, writing—review and editing. Xianlei Gao: investigation, writing—review and editing. Xia Wang: investigation, writing—review and editing. Rongkun Xu: writing—review and editing. Shangye Li: writing—review and editing. Lianlei Wang: supervision, project administration, funding acquisition. Xinyu Liu: conceptualization, supervision, project administration, funding acquisition.
